# Intelligent system based comparative analysis study of SARS-CoV-2 spike protein and antigenic proteins in different types of vaccines

**DOI:** 10.1186/s43088-022-00216-0

**Published:** 2022-03-07

**Authors:** Rabeb Touati, Ahmed A. Elngar

**Affiliations:** 1 LR99ES10 Human Genetics Laboratory, Faculty of Medicine of Tunis (FMT), University of Tunis El Manar, Tunis, Tunisia; 2 BIOPOLE Society, 63 Av. Hbib Bourguiba, Tunis, Tunisia; 3 Faculty of Computers and Artificial Intelligence, Beni-Suef University, Slah Salem Str., 62511 Beni-Suef City, Egypt; 4 American University in the Emirates, 503000, Dubai Intl. Academic City, Dubai, United Arab Emirates

**Keywords:** Coronavirus, Vaccines, Poliovirus, HIB, Hepatitis B, And PCV10, COVID-19, Genome signature, Genomic coding techniques

## Abstract

**Background:**

Coronaviruses, members of the Coronavirinae subfamily in the Coronaviridae family, are enveloped and positive-stranded RNA viruses that infect animals and humans, causing intestinal and respiratory infections. Coronavirus disease 2019 (COVID-19) is caused by a novel coronavirus, named Severe Acute Respiratory Syndrome Coronavirus 2 (SARS-CoV-2). This disease appeared, for the first time (December 2019), in China and has spread quickly worldwide causing a large number of deaths. Considering the global threat, the World Health Organization (WHO) has declared, in March 2020, COVID-19 as a pandemic. Many studies suggest the great effect of the existing vaccines to protect against symptomatic cases of death by the COVID-19 virus. This paper, proposes to compare the main antigenic proteins sequences of the existing vaccines with Spike (S) protein of the SARS-CoV-2 genome. Our choice of S protein is justified by the major role that plays it in the receptor recognition and membrane fusion process based on an intelligent system. Herein, we focus on finding a correlation between S protein and compulsory vaccines in the countries that have a less death number by COVID-19 virus. In this work, we have used a combination of coding methods, signal processing, and bioinformatic techniques with the goal to localize the similar patterns between the S gene of the SARS-Cov-2 genome and 14 investigated vaccines.

**Results:**

A total of 8 similar sequences which have a size more than 6 amino acids were identified. Further, these comparisons propose that these segments can be implicated in the immune response against COVID-19, which may explain the wide variation by country in the severity of this viral threat.

**Conclusions:**

Our in silico study suggests a possible protective effect of Poliovirus, HIB, Hepatitis B, PCV10, Measles, Mumps, and Rubella (MMR) vaccines against COVID-19.

## Background

Since December 2019, COVID-19, caused by the novel coronavirus SARS-CoV-2, has spread to more than 223 countries to date causing huge health and economic crisis [1–3]. The genome of SARS-CoV-2, like other families of Coronaviruses (CoVs), is an enveloped positive-sense single-stranded RNA virus. It belongs to the Coronaviridae family, the Orthocoronavirinae subfamily and the Betacoronavirus genus [1–3]. Coronaviruses (CoVs) can mainly infect mammals by Alpha (α) or Beta (β) coronaviruses, and predominantly can infect birds by Gamma (γ) or Delta (δ) Coronaviruses [4, 5]. The SARS-CoV-2 viral genome of 29,903 nucleotides, approximately, contains 5’and 3’ untranslated regions and 11 Open Reading Frames (ORFs) encoding 11 proteins including the S protein [6]. Probably, the modes of SARS-Cov-2 transmission among humans are via three primary pathways: inhaling respiratory droplets directly from infected persons, or contact with infected environmental surfaces know as “fomites” and touching your mucous membranes with soiled hands, or inhaling infected airborne particles. Recent researches indicate a high correlation between the SARS-COV-2 genome and the two genomes of the bat-CoV RaTG13 and the pangolin-CoV MP789 followed by two other genomes: CoVZC45 and CoVZXC21 [7, 8].

The outbreak of SARS-CoV-2 caused 2,797,435 deaths with a total of 127,847,262 confirmed cases (December 20, 2019 to March 29, 2021) that have variable damages from one country to another. In the majority of cases, huge damages were reported, such as in the USA, Brazil, and India causing respectively 562,526, 312,299 and 161,881, deaths as well as 30,962,803, 12,534,688, and 12,039,644 confirmed cases up to 29 March 2020 [9] Nevertheless, in other regions, such as, Laos, Vietnam, and Finland damages seem to be limited and the number of deaths, respectively, did not exceed 0, 35, and 822 [9]. However, for other regions, like, Nicaragua, Sierra Leone, Vietnam, and Madagascar, cases and damages seem to be limited and the number of deaths, respectively, did not exceed three hundred cases. These temporal variances in a number of case fatality rates can be caused by different factors: political and economic strategies, cultural behavior, age, and also health infrastructure, [10, 11]. Furthermore, the population’s immunological background is probably due to the vaccination strategies used in these countries [11–15]. From another point of view, different vaccines such as BCG (Bacillus Calmette-Guérin), OPV (Oral Poliovirus Vaccine), and MMR (Measles, Mumps, and Rubella vaccines) demonstrated an immune response to fight various pathogens [12, 14, 16, 17].

On the other hand, these different variations may be attributed to the adoption of a universal and long-standing BCG as again found to be very significantly protective for whom vaccination records were available [11, 18, 19]. In addition, the MMR vaccine protective potential was investigated based on S protein bioinformatic analysis [20]. Based on this computational biology analysis, the MMR vaccine was investigated as being potentially protective for adults and provides advantageous protection for children against COVID-19 as well. However, experimental analysis is required. Furthermore, pneumococcal vaccination PCV13 was again found to be very efficient in a study of 137,037 individuals who received SARS-CoV-2 PCR tests [21]. A recent study proves great similarities between the SARS-CoV-2 genome and pneumococcal vaccines PspA and PspC [22]. Indeed, other researchers found that polio, Hemophilus influenzae type-B (HIB), varicella, geriatric flu, MMR, PCV13, and hepatitis A / B (HepA-HepB) vaccines administered in the past 1, 2, and 5 years are associated with decreased SARS-CoV-2 infection rates [22, 23].

In this work, we propose in silico study to investigate the potential protective effect of 14 investigated vaccines (Bordetella Pertussis, Tetanus, Haemophilus influenzae type B (Hib), Corynebacterium Diphtheriae, Streptococcus pneumoniae, Hepatitis A, and Hepatitis B) against COVID-19. We aim to localize similar amino acid (aa) regions in the S protein of the SARS-CoV-2 genome and the main antigenic proteins in other vaccines which may lead to the production of cross-reactive antibodies against the target viruses as well as SARS-CoV-2. To achieve this goal, we used a combination of bioinformatics, and signal processing tools to identify the common amino-acid (aa) sequences of the main antigenic protein of SARS-CoV-2 and investigated vaccines.

## Methods

**Fig. 1:**
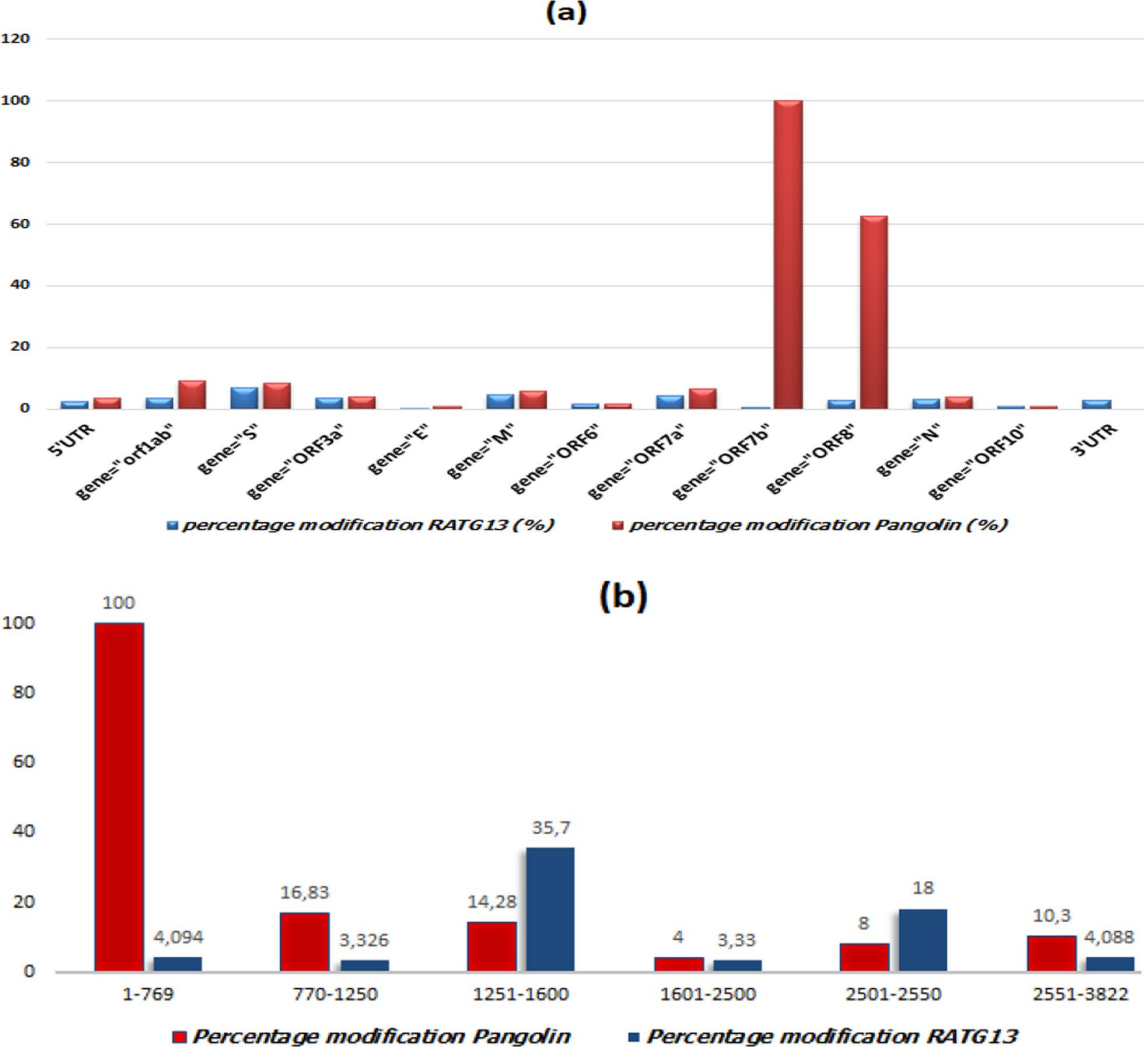
Percentage of nucleotide modifications of Sars-Cov-2 genome comparing to RATG13 and MP789 genomes [7]: **a** Percentage of nucleotide modifications of SARS-CoV-2 genome comparing to RATG13 and Pangolin genomes; **b** percentage of nucleotide modifications of S gene of SARS-CoV-2 genome comparing to the S genes of RATG13 and Pangolin genomes [7]

Recent research [7] has been suggested the SARS-Cov-2 genome shares 96% genetic similarity with a RATG13 coronavirus genome and 86% genetic similarity with the pangolin coronavirus genome. Subfigure (a) of Fig. 1 presents the distribution of nucleotide modifications by percentage along the SARS-COV-2 region compared to the RATG13 and the MP789 coronavirus genomes. Spike protein presents high recombination between RATG13 and the MP789 coronavirus genomes as shown in subfigure (a) of Fig. 1. Specifically in the position 1251 to 1600 base pairs, the total nucleotide mutation is equal to 14.28% (50 nucleotides) between the two coronavirus genomes (the pangolin and the SARS-CoV-2). These mutations are less than the mutation located between bat coronavirus and SARS -CoV-2 S gene (35% and 125 modified nucleotides). This research [7] suggests that the SARS-CoV-2 genome is the result of recombination events between two coronavirus genomes: The bat-CoV RATG13 and the pangolin-CoV MP789.

**Fig. 2:**
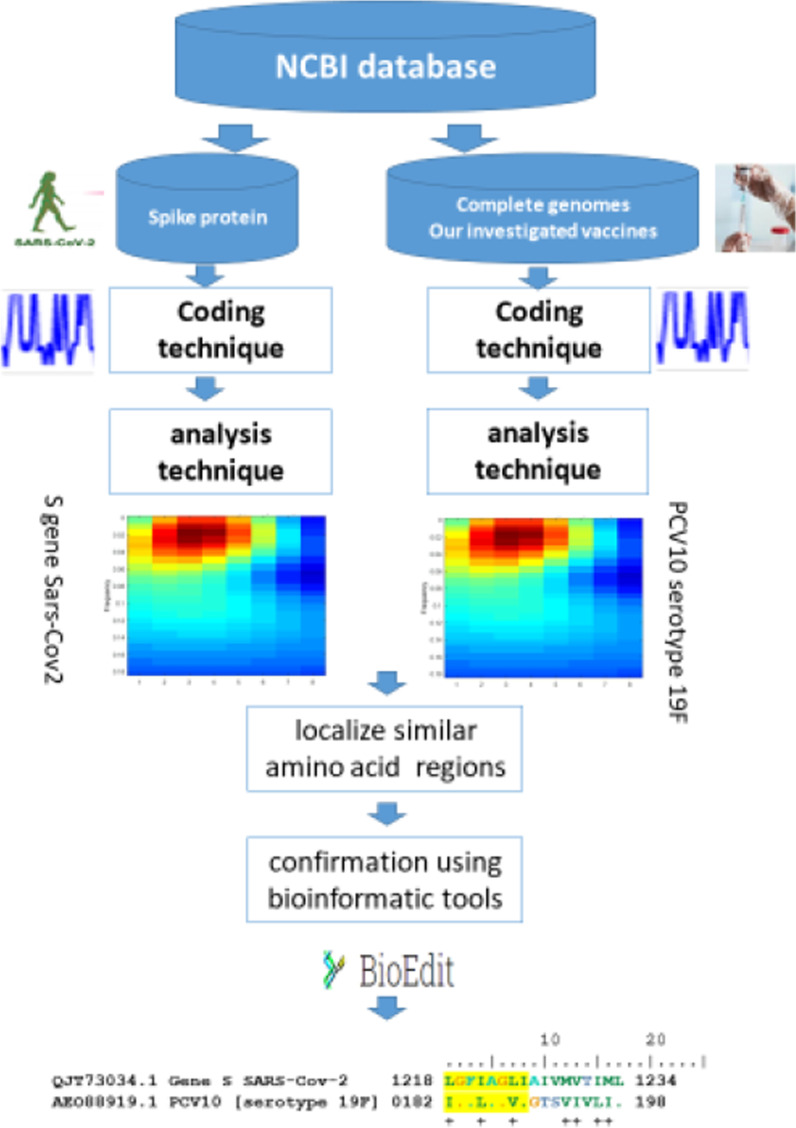
Flowchart diagram of methodology to localize the similarities between two amino acid sequences

Figure 2 presents the Flowchart diagram of our adopted localization methodology to find similar amino acid sequences between SARS-CoV-2 genome and our investigated sequences. Here we have used the recombination between bioinformatics techniques and signal processing tools.

### Vaccines and Sequences investigated (databak accession numbers)

Our study is focused on the vaccines included in one of the countries presenting a very low number of confirmed COVID-19 cases. Our investigated vaccines include main old and more recent vaccines (a number equal to 14) with 34 protein sequences: BCG, Poliovirus, Measles, Mumps, Corynebacterium diphtheria, Tetanus, and Bordetella pertussis vaccines and more recent vaccines against hepatitis B and A viruses, Rubella virus, Hemophilus influenzae type B (Hib) and Streptococcus pneumoniae (PCV10, pspC, protein PspC, Protein A) [24]. The amino-acid sequences of these antigenic proteins (n = 34) constituting those vaccines as well as the amino acid sequence of Spike protein of the Wuhan Sars-Cov-2 genome strain were obtained from the database NCBI Genbank (https://www.ncbi.nlm.nih.gov). Accession numbers are presented in Table 1.

**Table 1:** Investigated vaccines and their corresponding antigenic proteins obtained from NCBI Genbank (https://www.ncbi.nlm.nih.gov)

	Vaccine	Protein	Accession N°		Vaccine	Protein	*Accession N°*
1	*Tetanus*	Toxin protein	AAA23282.1	11	*Hemophilus influenzae serotype B (Hib)*	Capsulation protein	CWW30252.1
2	*Corynebacterium diphtheriae*	Toxin protein	CAA00374.1			Capsular polysaccharide biosynthesis protein	WP_015702013.1
3	*Hepatitis B*	HBsAg-adw2	AAW65557.1	12	*Poliovirus*	VP1 protein: Sabin 1 strain	AAL89597.1
		HBsAg-adr	AAW65588.1			VP1 protein: Sabin 2 strain	AAL92486.1
4	*Bordetella pertussis*	Toxin protein	AQW64178.1			VP1 protein: Sabin 3 strain	AAL89592.1
5	*Mumps*	Hemagglutinin/neuraminidase protein	ACN50032.1	13	*Streptococcus pneumoniae (PCV10)*	Capsular polysaccharide/biosynthesis protein (serotype 1)	COS99248.1
		Fusion protein	ACN50030.1			Capsular polysaccharide/biosynthesis protein (serotype 4)	AAK20668.1
6	*Rubella*	Polyprotein E1/E2	ACN50046.1			Capsular polysaccharide/biosynthesis protein (serotype 5)	CAI32793.1
7	*Hepatitis A*	VP1 protein	AAA45466.1			Capsular polysaccharide/biosynthesis protein (serotype 6B)	AAK20683.1
		VP3 protein	AAA45466.1			Capsular polysaccharide-biosynthesis protein (serotype 7F)	CAI32924.1
8	*Measles*	Fusion protein	AAF85704.1			Capsular polysaccharide/ biosynthesis protein (serotype 9 V)	CAI33023.1
		Hemagglutinin protein	AAF85705.1			Capsular polysaccharide/ biosynthesis protein (serotype 14)	CAI33319.1
9	*Bacillus Calmette-Guérin (BCG)*	Immunogenic protein MPB64	AIC33023.1			Capsular polysaccharide/ biosynthesis protein (serotype 18C)	CAI33577.1
		Immunogenic protein MPB70	BAA07402.1			Capsular polysaccharide biosynthesis protein (serotype 19F)	AEO88919.1
		Immunogenic protein MPB83	BAA11027.1			Capsular polysaccharide/ biosynthesis protein (serotype 23F)	AAC69522.1
10	*Streptococcus Pneumoniae;* *Pneumococcal Surface Protein A*	PspA Q9LAZ1	AAF27700.1	14	*Streptococcus Pneumoniae;* *pspC, Pneumococcal Surface protein PspC*	Q9KK40	AAF73787.1
		PspA O34097, pspA,	AAC62252.1				
		PspA Q9LAY4	AAF27707.1				

### Amino acid sequence alignment and hot spot analysis

**Fig. 3:**
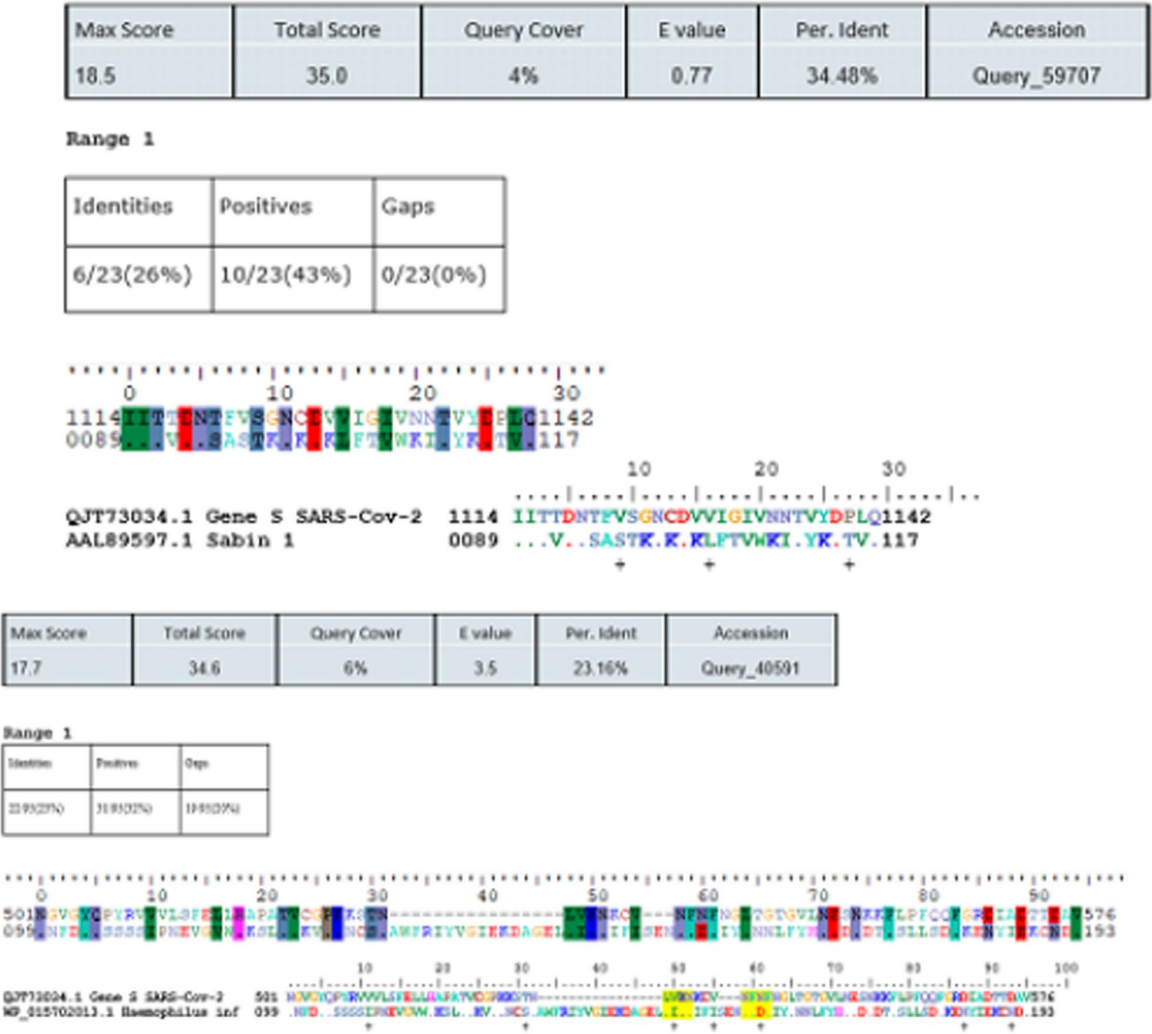
Example of Bioedit ((http://www.mybiosoftware.com/bioedit-7-0-9-biological-sequence-alignment-editor.html) version 7.2.5) results to identify similar pattern located between S protein of SARS-CoV-2 genome and two protein sequences of vaccines corresponding of two viruses (Poliovirus and Hemophilus influenzae serotype B (Hib))

We aim to detect similar amino-acid (aa) sequences between protein sequences. The presented results were obtained using Blastp program. This program can detect identical amino-acids and, or similar amino-acids of two genomic sequences. These results were presented using BioEdit software (version 7.2.5) (http://www.mybiosoftware.com/bioedit-7-0-9-biological-sequence-alignment-editor.html). Figure 3 presents an example of our obtained results after comparing the S protein of the Sars-Cov-2 genome and two sequences of the tetanus vaccine. These obtained results present different similar patterns with their position in each genome.

### Numerical mapping of amino acid sequences

The conversion of the genomic sequences into numerical ones using signal processing tools is an important step to characterize the unknown region [25–28]. It allows rapid image observation of similar patterns before assessing more precise analysis. By applying the Electron–Ion Interaction Potential (EIIP) coding technique, we can obtain for each protein sequence a signal. This type of coding technique (EIIP) has been used to transform the amino acid (aa) sequence into numerical representation [25]. For that, we converted our amino-acid sequences into numerical ones using the following Table 2, where each amino acid was represented by its corresponding value of the EIIP coding technique.

**Table 2:** EIIP coding technique for transformation of the amino acid sequence into a signal

Amino acid	Single letter symbol	EIIP	Amino acid	Single letter symbol	EIIP
Ala	A	0.0373	Leu	L	0
Arg	R	0.0959	Lys	K	0.0371
Asn	N	0.0036	Met	M	0.0823
Asp	D	0.1263	Phe	F	0.0946
Cys	C	0.0829	Pro	P	0.0198
Gln	Q	0.0761	Ser	S	0.0829
Glu	E	0.0058	Thr	T	0.0941
Gly	G	0.005	Trp	W	0.0548
His	H	0.0242	Tyr	Y	0.0516
Ile	I	0	Var	V	0.0057

**Fig. 4:**
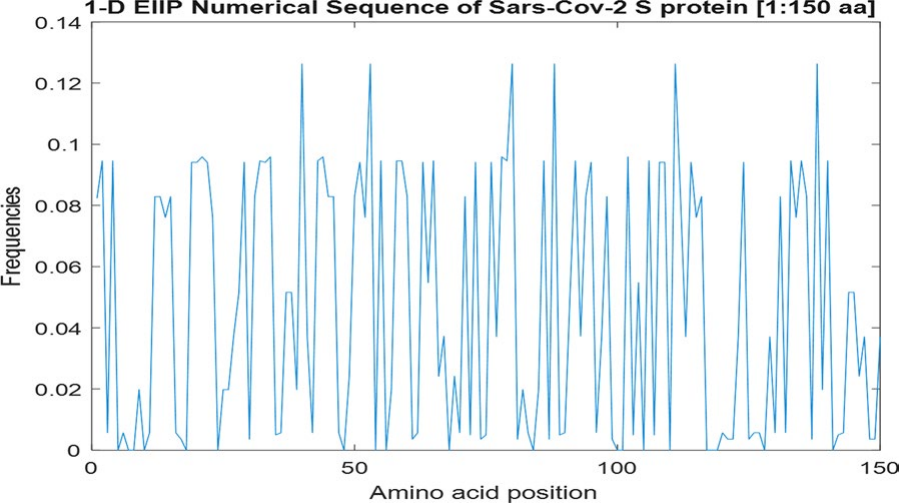
EIIP Numerical representation (generated signal) of 150 amino acids of Sars-Cov-2 Spike protein

Figure 4 shows the 1-D signal representation of protein sequence corresponding to 150 amino acids of S-protein of Sars-Cov-2 genome. Each signal value corresponds to an amino acid position in the protein sequence.

After transforming the amino-acid sequence into a signal, we aimed to see it in a 2-D representation to focus more on the information that can contain. The Continuous Wavelet Transform (CWT) (along 64 scales with *w*_0_ ~ 5.5) was applied to protein signal to obtain a protein image (2-D scalogram) [27, 28].

$$w_{0} = 2*\pi *f_{0}$$

$w_{0}$ corresponds to the oscillations number of wavelet transform, and the parameter f_0_ is the central frequency of the basic wavelet. These images (wavelet scalograms) are the best method to detect the homologous regions between two amino-acid sequences [27, 28].

## Results

### Numerical mapping

To transform a protein sequence into numerical ones, we need to apply a coding technique to have a signal and an analysis technique to have an image.

**Fig. 5:**
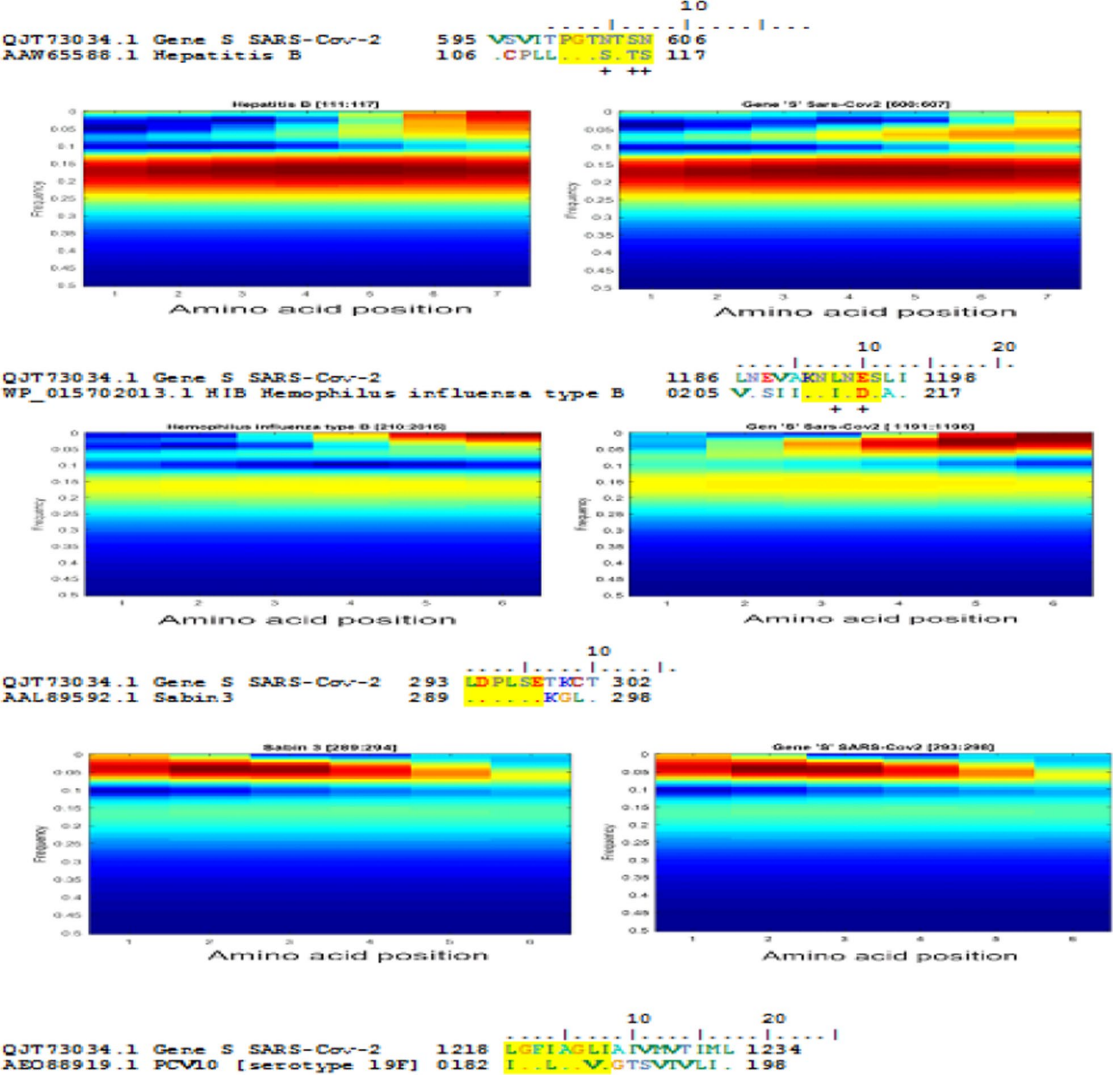
Wavelet representation (Scalograms) of similar regions (patterns) of amino acid region of SARS-COV-2 S gene (QJT73034.1) compared with the acid amino vaccines; the first column shows the S gene of the SARS-cov-2 genome and the second shows the vaccine sequence

In this work, and after applying different methods, we have chosen to use the EIIP as a coding technique and the wavelet analysis as an analysis technique. After applying the EIIP coding technique and CWT of an amino acid sequence, we obtained a scalogram image (modulo of the matrix contains CWT coefficients). The amino acid scalograms (2D) are the best way to see the similarities between two sequences if it exists. As a result, we obtained two similar patterns (scalograms) that confirm these similarities. Figure 5 presents images corresponding to similar patterns between the region of S protein of Wuhan SARS-CoV-2 genome and some regions of our investigated amino-acid sequences.

### Alignment of amino acid sequences

**Fig. 6:**
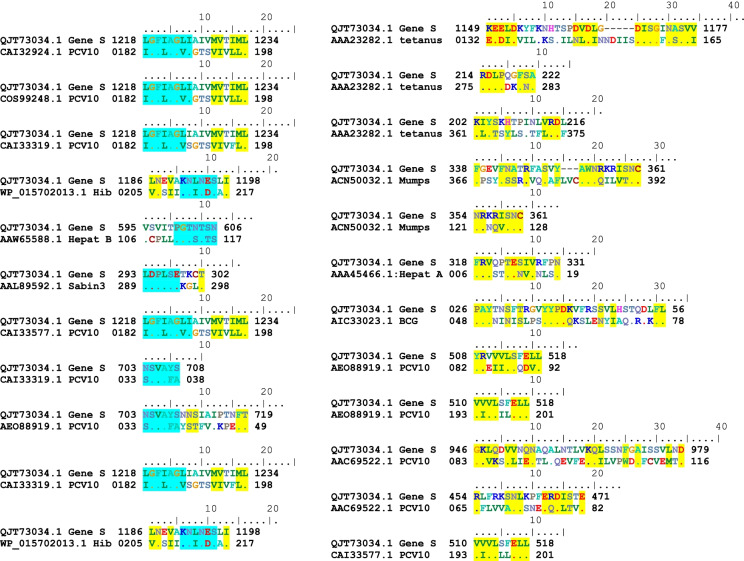
Highly similar sequences were identified in our investigated vaccines and SARS-CoV-2 proteins using BioEdit software (http://www.mybiosoftware.com/bioedit-7-0-9-biological-sequence-alignment-editor.html)

As a result, similar segments (4–8 aa) between the S protein of the SARS-Cov-2 genome and our investigated vaccine antigenic proteins were identified (Fig. 6 and Table 3). Only antigenic protein included in Hepatitis B, Hib, Poliovirus, PCV10 vaccines showed 6 to 8 similar consecutive amino-acids. The corresponding motifs are indicated in Table 3. PCV10 antigenic proteins shared 2 similar motifs (  ) of 8 and 6 amino acids.

**Table 3:** Results of total similar patterns number were identified in our investigated vaccines and SARS-CoV-2 proteins

Vaccine	Protein	% Identity	Number of similar patterns less than 6 aa and more or equal to 2	Number of similar patterns more or equal to 6 aa	Pattern more than 6 aa
Tetanus	Tetanus toxin protein	35.29	47	0	–
Corynebacterium diphtheriae	Diptheria toxin protein	45.00	11	0	–
Hepatitis B	HBsAg-adw2	55.56	5	0	–
	HBsAg-adr	50.00	5	**1**	
Bordetella pertussis	Bordetella pertussis toxin protein	23.26	6	0	–
Measles	Measles hemagglutinin protein	23.75	4	0	–
	Measles fusion protein	45.45	2	0	–
Rubella	Rubella polyprotein E1/E2	NONE	–	–	–
Mumps	Mumps xxx protein	31.43	9	0	–
	Mumps hemagglutinin/neuraminidase protein	27.14	32	0	–
Hepatitis A	Hepatovirus A VP1 protein	34.29	22	0	–
	Hepatovirus A VP3 protein	50.00	2	0	–
Bacille Calmette-Guérin (BCG)	Immunogenic protein MPB83	27.27	2	0	–
	Immunogenic protein MPB70	52.17	4	0	–
	Immunogenic protein MPB64	35.48	10	0	–
*Hemophilus influenzae* type B (Hib)	Capsulation protein	25.00	5	0	–
	Capsular polysaccharide biosynthesis protein	23.16	7	1	
Poliovirus	VP1 protein (Sabin 1 strain)	34.48	7	0	–
	VP1 protein (Sabin 2 strain)	42.11	4	0	–
	VP1 protein (Sabin 3 strain)	26.67	0	1	
*Streptococcus pneumoniae* (PCV10)	Capsular polysaccharide biosynthesis protein [serotype 19F]	23.15	18	2	
	Capsular polysaccharide biosynthesis protein [serotype 23F]	26.47	6	0	–
	Capsular polysaccharide biosynthesis protein [serotype 18C]	35.29	15	2	
	Capsular polysaccharide biosynthesis protein [serotype 14]	27.38	29	2	
	Capsular polysaccharide biosynthesis protein [serotype 9 V]	43.75	11	0	–
	Capsular polysaccharide biosynthesis protein [serotype 7F]	35.29	31		
	Capsular polysaccharide biosynthesis protein [serotype 6B]	43.75	6	0	–
	Capsular polysaccharide biosynthesis protein [serotype 5]	29.76	20	**1**	
	Capsular polysaccharide biosynthesis protein [serotype 1]	35.29	30	**2**	
	Capsular polysaccharide biosynthesis protein [serotype 4]	35.29	23	**1**	

Figure 6 contains 23 similar patterns corresponding to more of 4 similar consecutive amino-acids of the S protein and our investigated vaccines. A total of 11 similar patterns have a size equal or more than 6 amino acids were identified.

Table 3 presents the total repetition number of each similar pattern that was identified between vaccine and S protein of Sars-Cov-2 genome.

**Fig. 7:**
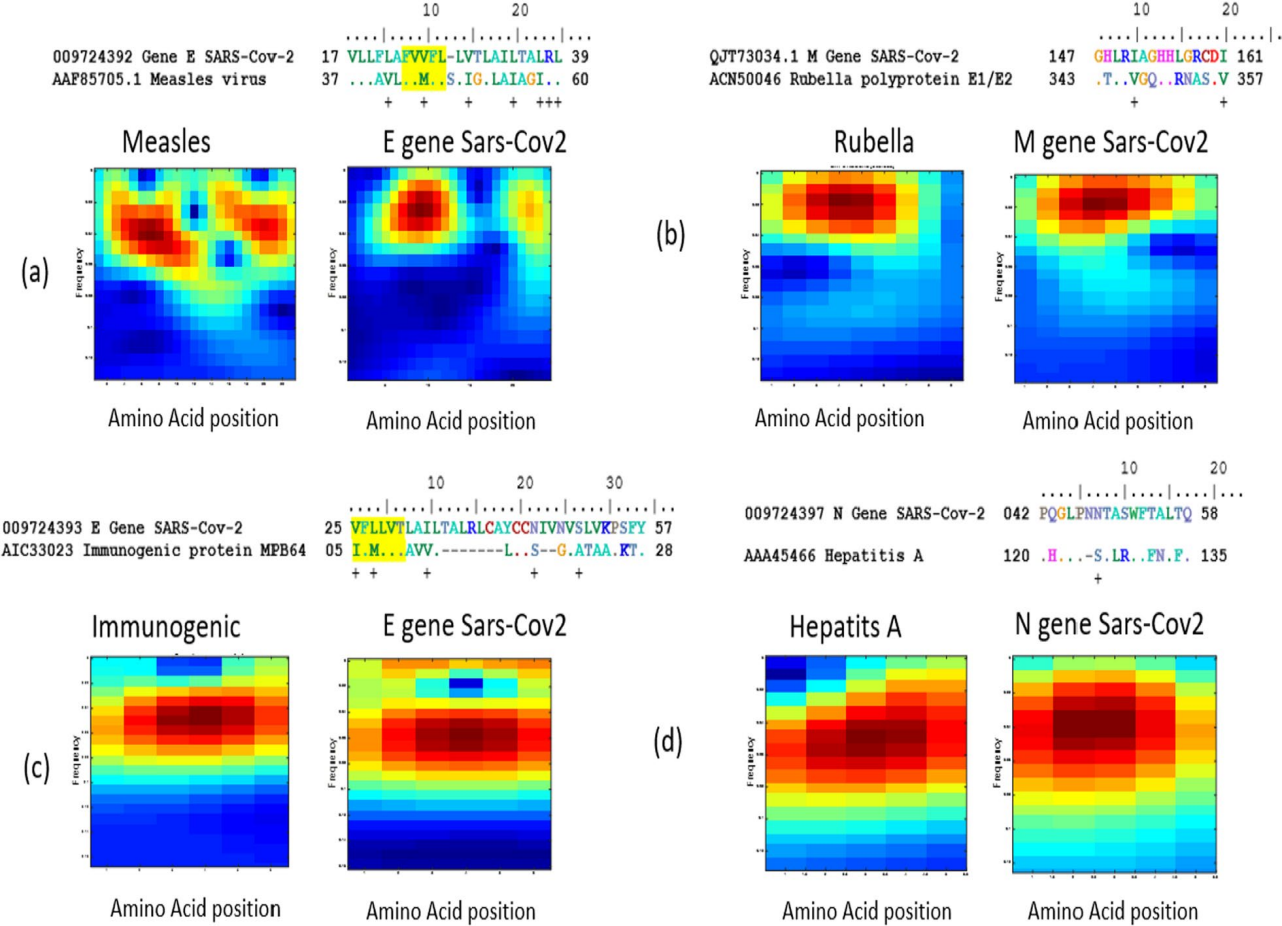
Scalograms (amino acids Wavelet images) of different amino acids region of different genes of SARS-COV-2 genome compared with the acid amino vaccines

To more see if these patterns are present in the other genes of the SARS-Cov-2 genome, we were also able to map and compare all other SARS-CoV-2 genes with our investigated vaccines. We confirm that these presented similar patterns (  ,  ,  , and  ) between the Sars-Cov-2 S gene and vaccines are not presented in the other genes of the Sars-Cov-2 genome. Moreover, we detect a new similar pattern ‘  ’ between the BCG vaccine and Sars-Cov-2N gene (Fig. 7).

## Discussion

Different researchers prove that the Sars-Cov-2 genome is the result of the recombination between two beta-coronavirus genomes: Pangolin and Bat coronavirus [7, 8, 29, 30]. Various in silico studies search for the effect of the vaccination, especially with pneumococcal vaccines, to protect against symptomatic cases of SARS-CoV-2 infection and death [31–36]. Our main objective was to find similar amino-acid sequences in the S protein of the Sars-Cov-2 genome and the investigated proteins sequences mentioned above using the recombination between bioinformatics and signal processing tools. This study using an intelligent system suggests the possible influence of Hepatitis B, Hib, Poliovirus, Tetanus, and PCV10 vaccines in protecting against COVID-19. In addition, this work could explain the variation in the number of deaths among countries and the possibility that these vaccines contain antigens that might be cross-reactive with SARS-CoV-2. For this, we consider that some of these investigated vaccines as real supportive protective measures in the fight against the COVID-19 pandemic. A recent review [37] presents a comparative table that contains multiple vaccine platforms and has been explored to develop the COVID-19 vaccine. Each vaccine platform has unique advantages and disadvantages. Most of these vaccines use either the Spike protein or its receptor-binding domain (RBD) as the antigen.

For this, we studied the effect of our investigated vaccines and see if it can be implicated to boost our immune system against COVID-19. We investigate the possibility of the protection against COVID-19 through inducing a cross-reactive antibody by different existing vaccines. To achieve this goal, we used a combination of bioinformatic and signal processing tools to compare the amino-acid sequences of the main S protein of SARS-CoV-2 genome and our investigated vaccines. A numerical mapping showed two similar patterns corresponding to an exposed site have a size of 7 and 8 amino-acids: ‘  ’ detected in HBsAg-of Hepatitis B vaccine and ‘  ’ detected in PCV10 vaccine. Two other patterns (‘  ’ and ‘  ’) in two investigated vaccines (Polio, PCV10,) are identified in the S gene of the SARS-CoV-2 genome. Given the novel aspects of the importance of Hepatitis B, Streptococcus pneumoniae, and Polio immunities as potential protective factors in the COVID-19 pandemic, measures to organize mass immunization against these viruses should be strengthened. Vaccination with attenuated viruses may explain partially the low symptomatic infection rate among children as there are other factors yet to be considered.

## Conclusions

The main of this work is tracking the effect of existing vaccines against COVID-19 threats using suitable computational techniques. As a conclusion, our obtained results indicate that some antigenic proteins in these investigated vaccines may protect against severe COVID-19. We suggest that these types of vaccines can reduce the risk of COVID-19 mortality, but we note that this silico study must be confirmed using in vitro analysis.

## Data Availability

All data generated or analyzed as part of this study are included in this published article.

## Abbreviations

CoV: Coronaviruse
CoVs: Coronaviruses
COVID-19: Coronavirus disease 2019
SARS-CoV-2: Severe acute respiratory syndrome coronavirus 2
S: Spike
ORFs: Open reading frames
MMR: Measles, mumps, and rubella
BCG: Bacillus Calmette-Guérin
OPV: Oral poliovirus vaccine
HIB: Hemophilus influenzae type-B
CWT: Continuous wavelet transform
EIIP: Electron ion interaction potential
PCV10: Streptococcus pneumonia
NCBI: National Center for Biotechnology Information
WHO: World Health Organization
BLAST: Basic local alignment search tool
Blastp: Protein blast
